# Genetic sex determination of mice by simplex PCR

**DOI:** 10.1186/s13293-017-0154-6

**Published:** 2017-10-17

**Authors:** Simon James Tunster

**Affiliations:** Centre for Trophoblast Research, Department of Physiology, Development and Neuroscience, University of Cambridge, Cambridge, CB2 3EG UK

**Keywords:** Sex genotyping of mice, Simplex PCR, *Rbm31x*, *Rbm31y*

## Abstract

**Background:**

Investigating fetal development in mice necessitates the determination of fetal sex. However, whilst the sex of adult and juvenile mice can be readily distinguished from anogenital distance, the sex of fetal and neonatal mice cannot be identified visually. Instead, genetic sex must be determined by PCR amplification of X chromosome genes with divergent Y chromosome gametologs. Existing simplex PCR methods are confounded by small size differences between amplicons, amplification of unexpected products, and biased amplification of the shorter amplicon.

**Results:**

Primers were designed flanking an 84 bp deletion of the X-linked *Rbm31x* gene relative to its Y-linked gametolog *Rbm31y*. A single product was amplified from XX samples, with two products amplified from XY samples. Amplicons were resolved by gel electrophoresis for 20 min, with unbiased amplification of both products observed in XY samples.

**Conclusion:**

This method achieves rapid and unequivocal genetic sex determination of mice in low volume PCR reactions, reducing reagent usage and simultaneously eliminating shortcomings of previous methods.

## Background

A short gestation and large litter size make the laboratory mouse a valuable and widely used research tool for studying fetal and placental development. However, sexual dimorphism in basal gene expression and placental phenotypes necessitates determination of fetal sex in such studies [1, 2]. Whilst the sex of adult and juvenile mice can be readily distinguished by comparing the anogenital distance, accurately identifying neonatal sex is more problematic [3], whereas fetal sex cannot be identified visually. Instead, genetic sex must be determined by PCR. The first such method amplified the Y chromosome genes *Sry* or *Zfy*, with amplification of the X chromosome microsatellite repeat *DXNds3* serving as an amplification control [4]. Subsequent variations to the protocol have targeted different regions of *Sry* or *Zfy*, or used alternative autosomal or X chromosome genes as amplification controls [5–9]. Such methods rely on successful amplification of the Y chromosome to identify XY animals, with XX animals inferred by the absence of amplification from the Y chromosome. However, these methods necessitate multiplex PCR reactions in which two sets of primers are used to amplify the two targets in the same reaction, presenting difficulties not encountered in simplex PCR methods [10, 11].

More recently, simplex PCR methods have been developed that exploit differences between divergent X-linked genes and their Y-linked gametologs (Table 1). Amplification of the *Uba1*/*Uba1y* genes exploits a 19 bp deletion in the Y-linked *Uba1y* [12], whereas amplification of *Kdm5c*/*Kdm5d* exploits a 29 bp deletion in the Y-linked *Kdm5d* [13]. However, these methods rely on differentiating between similarly sized PCR products, necessitating lengthy gel run times (> 45 min) on high percentage agarose gels (> 2%). Furthermore, the *Uba1y* forward primer contains mismatched bases to the target sequence, as do both *Kdm5c* primers. More recently, a third method has been described that amplifies the *Xlr* and *Sly* genes, with predicted products of 685 bp (*Xlr*) and 280 bp (*Sly*) [14]. Whilst the 405 bp size difference between products should readily distinguish fetal sex, additional unexpected products of ~ 660 and ~ 480 bp were amplified from the X chromosome in XX samples, with a failure to amplify *Xlr* in XY samples resulting from biased amplification of the shorter *Sly* amplicon. Thus, this method results in the somewhat counter-intuitive amplification of three products in XX samples and a single product in XY samples. Whilst the existing methods can be used to successfully determine fetal sex, a rapid and unambiguous simplex-PCR method has yet to be developed. Ideally, such a method would generate products that are sufficiently similar in size to prevent biased amplification of the smaller product, whilst ensuring suitable difference in size to facilitate rapid resolution by gel electrophoresis.

**Table 1:** Summary of existing simplex PCR methods for determining genetic sex of mice

Targets	Predicted products	Observed products	Potential limitation	Reference
*Uba1* (X) *Uba1y* (Y)	217 bp (X) 198 bp (Y)	XX 217 bp XY 198 bp; 217 bp	Forward primer is mismatched to *Uba1y*. Small difference (19 bp) in amplicon size necessitates extended gel run time.	[12]
*Kdm5c* (X) *Kdm5d* (Y)	331 bp (X) 302 bp (Y)	XX 331 bp XY 331 bp; 302 bp	Primers are mismatched to *Kdm5c*. Small difference (29 bp) in amplicon size necessitates extended gel run time.	[13]
*Xlr* (X) *Sly* (Y)	685 bp (X) 280 bp (Y)	XX 685 bp, ~ 660 bp, ~ 480 bp XY 280 bp	Additional products amplified from X chromosome. Failure to amplify X chromosome in XY samples.	[14]

The three existing methods of genetic sex determination of mice by simplex PCR are summarized, detailing expected and observed PCR products and the potential limitations of each method

The recently sequenced male-specific region of the mouse Y chromosome identified additional X chromosome genes with acquired and amplified Y chromosome gametologs [15], providing the opportunity to explore potential novel targets to be utilized in a simplex PCR-based method of sex genotyping. Many of the acquired Y chromosome genes belong to large gene families, with dozens, or even hundreds, of Y chromosome copies, and estimates of up to 25 X-chromosome gametologs [15]. This amplification and subsequent divergence of family members explains the amplification of additional unanticipated products from *Xlr* [14]. Unique amongst the annotated acquired Y-chromosome genes is the two-copy Y-linked *Rbm31y* and the single copy X-linked *Rbm31x*. The low copy number of these gametologs mitigates the risk of divergent family members yielding amplification of additional products. Sequence alignment of *Rbm31x* and *Rbm31y* identified a high degree of sequence homology and revealed an 84 bp deletion in *Rbm31x* compared with *Rbm31y*. This work reports the development and testing of a new method of sex determination of fetal mice by amplification of the *Rbm31x/y* genes.

## Methods

**Fig. 1:**
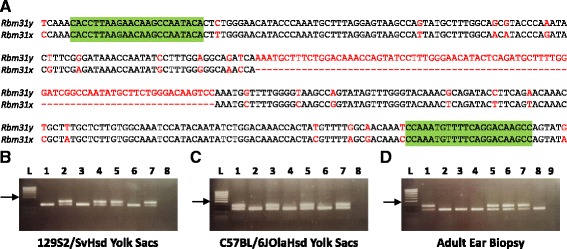
Genetic sex determination of mice by amplification of *Rbm31y* and/or *Rbm31x* by simplex PCR. **a** Alignment of *Rbm31y* (nucleotides 997–1411) and *Rbm31x* (nucleotides 990–1320). Sequences highlighted in red denote mismatches and the 84 bp deleted region. Primers highlighted in green. Sex determination by simplex PCR of yolk sac lysates from samples of 129S2/SvHsd (**b**) and C57BL/6JOlaHsd (**c**) genetic backgrounds. Lane L = PCR Ranger 100 bp Ladder (GeneFlow); Lanes 1–7 = yolk sac lysates; Lane 8 = no template control. **d** Confirmation of methodology using ear biopsies of adult animals from 129S2/SvHsd (129) and C57BL/6JOlaHsd (BL6) backgrounds. Lane L = PCR Ranger 100 bp Ladder (GeneFlow); Lanes 1–8 = ear biopsy lysates: XY 129, XX 129, XX BL6, XX 129, XY 129, XY BL6, XY 129, XX BL6; Lane 9 = no template control. Arrows indicate 500 bp band

The single exon of *Rbm31x/Gm4916* (XM_003085289) is 1775 bp in length, whereas that of *Rbm31y* (NM_028970.1) is 1958 bp in length. Transcript alignment using the EMBL-EBI Pairwise Sequence Alignment Tool [16] confirmed 83.9% sequence identity and revealed an 84 bp deleted region in *Rbm31x*, corresponding to nucleotides 1149–1232 of *Rbm31y* (Fig. 1a). A second 16 bp deleted region in *Rbm31x* was also identified, corresponding to nucleotides 1748–1763 of *Rbm31y*. Primers were designed using Primer3 to amplify homologous regions flanking the 84 bp deletion (Forward: CACCTTAAGAACAAGCCAATACA; Reverse: GGCTTGTCCTGAAAACATTTGG) to yield a 269 bp product from the X chromosome and a 353 bp product from the Y chromosome. Primer specificity was confirmed by in silico PCR [17] prior to being tested on yolk sac and ear biopsy lysates.

Archived yolk sacs and ear biopsies from 129S2/SvHsd and C57BL/6JOlaHsd strains (Envigo) had been previously lysed by incubation overnight at 55 °C in 450 μl (yolk sacs) or 100 μl (ear biopsies) lysis buffer (50 mM Tris (pH 8), 10 mM EDTA, 20 mM NaCl and 0.031% SDS) supplemented with 400 μg/ml Proteinase K (Promega). Lysates were vortexed, heated at 95 °C for 15 min, cooled for 10 min and 0.6 μl used as template in a 15 μl PCR reaction containing 1X buffer, 0.2 mM each dNTP (ThermoFisher), 0.17 μM each primer (Sigma), and 0.5U DreamTaq HotStart DNA Polymerase (ThermoFisher). Thermocycler conditions were 94 °C for 2 min, followed by 30 cycles of 94 °C for 20 s, 60 °C for 20 s and 72 °C for 30 s, with a final elongation at 72 °C for 5 min. Reactions were run on a MyCycler (Bio-Rad), with a total run time of under 70 min. PCR reactions were mixed with 6X Orange G Gel Loading Dye, loaded on to a 1% (*w*/*v*) agarose gel containing 10 μl SafeView (NBS Biologicals), electrophoresed in 1X TAE (40 mM Tris acetate, 2 mM Na_2_EDTA) at 10 V/cm for 20 min and visualized on a UV transilluminator. Fetal sex was confirmed using *Uba1/Ube1y1* primers as described previously [12].

## Results and discussion

The *Rmb31x/y* method yielded the anticipated results from yolk sac samples from both 129S2/SvHsd and C57BL/6JOlaHsd strain backgrounds, with two products of 269 and 353 bp produced in male samples with only the 269 bp product produced in female samples (Fig. 1b, c), with results confirmed using primers for *Uba1*/*Uba1y* [12]. The methodology was further validated by performing the PCR assay in a blinded manner on ear biopsies from adult animals from both 129S2/SvHsd and C57BL/6JOlaHsd backgrounds for which sex had been determined visually (Fig. 1d). The visually determined sex was confirmed by the genetically determined sex for all samples tested.

Further to facilitating rapid, unambiguous genetic sex determination of mice, this method also achieves substantial savings in reagent usage. The ability to use lysates directly as template eliminates the need to purify genomic DNA prior to PCR. The low volume (15 μl compared with typical 25 μl reaction volume) PCR reactions also reduce reagent usage including primers, dNTPs, and Taq polymerase. Furthermore, the ability to resolve PCR reactions on 1% gels as opposed to 2% gels further saves on reagents.

In summary, this method facilitates rapid genetic sex determination of mice from lysed samples within 2 h, substantially reducing reagent usage and yielding results that are unambiguous and simple to interpret.
